# Cocos: Constructing multi-domain protein phylogenies

**DOI:** 10.1371/currents.RRN1240

**Published:** 2011-06-09

**Authors:** Max Homilius, John Wiedenhoeft, Sebastian Thieme, Christoph Standfuß, Ivan Kel, Roland Krause

**Affiliations:** ^*^Dept. of Computer Science, Free University of Berlin, Germany; ^†^Department of Computational Molecular Biology, Max Planck Institute for Molecular Genetics, Berlin, Germany and ^‡^Department of Vertebrate Genomics, Max Planck Institute for Molecular Genetics Berlin, Free University of Berlin, Germany

## Abstract

Phylogenies of multi-domain proteins have to incorporate macro-evolutionary events, which dramatically increases the complexity of their construction.

We present an application to infer ancestral multi-domain proteins given a species tree and domain phylogenies. As the individual domain phylogenies are often incongruent, we provide diagnostics for the identification and reconciliation of implausible topologies. We implement and extend a suggested algorithmic approach by Behzadi and Vingron (2006).

## Introduction

Domains characterize proteins structurally, evolutionarily and functionally [1]. More than half of the proteins in prokaryotes and about 80 percent of the proteins in eukaryotes are composed of multiple domains [2]. About 200 domains in eukaryotes occur in diverse architectures [3] and provide a challenge for phylogenetic inference, as proteins can be composed of non-homologous elements. Evolutionary events such as the fusion of proteins or the loss of domains need to be considered in phylogenetic analyses of multi-domain proteins (MDPs). Behzadi and Vingron put forward an iterative procedure (BV) to reconstruct ancestral domain compositions using the phylogenetic relationship within domain families [4]. See Figure 1 for an overview. Their algorithm minimizes the number of the macro-evolutionary events *protein fusion* and *domain loss* using a set-theoretic formulation and is independent of the order of the domains in the proteins.

**Figure:**
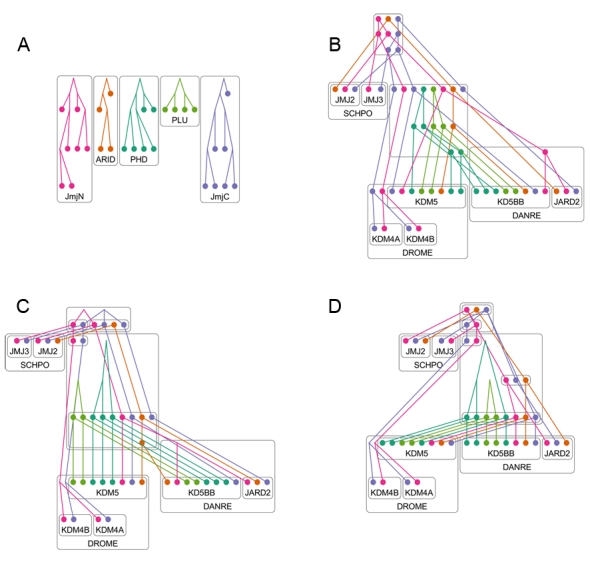
**Figure 1. Example and principal approach. **Reconstruction of the ancestral domain composition for JMJ-associated proteins in *Drosophila melanogaster, Schizosaccharomyces pombe* and *Danio rerio*. (A) Phylogenies are inferred for each domain. (B) Domain trees are embedded in the species tree. (C) Domains are partitioned into proteins. (D) Inconsistencies in the domain trees are curated.

 The algorithm consists of the following steps: 

1. Input a species tree, a set of domain trees and extant domain compositions, i.e. a partition of the domain trees' leaf set.
2. Recursively map domain tree nodes to species tree nodes at the least common ancestor (LCA) of all child domain nodes, starting at the leaf level.
3. Recursively walk through the species tree nodes (bottom-up). In all child species, the domains are already partitioned. Establish correspondence between domain nodes in the child species and those in the current species (relabeling of domain nodes), then find an optimal partition in the current species which is closest to all child partitions in terms of the weighted number of fusions and deletions.

 


Previous analyses of MDPs have decided against the use of phylogenetic trees for domains [5] or relied on establishing phylogenies only for domain trees with high internal bootstrap support [6]. Recently, an alternative approach for the reconstruction of MDPs including domain trees was proposed [7].

We implemented BV, tested it and and identified critical issues that need to be addressed for successful reconstruction of phylogenies of MDPs using BV. Due to the large number of possible domain combinations a good set of partitions cannot be found by brute-force enumeration. We implemented a heuristic called weak edge erosion,  which  yields  close to optimal solutions faster than simulated annealing suggested by [4]. In the practical application it showed that most domain trees are incongruent to each other and the species tree. We implemented a simple procedure to detect and rectify problematic cases.

In the following, we present our findings in detail, provide an implementation and show how to use it in practice. First, BV and the individual improvements are introduced formally.

## Methods   

### The algorithm by Behzadi and Vingron

The algorithm BV uses the information of domain trees and the composition of extant proteins to infer the domain composition of ancestral proteins [4]. The original publication contains a worked example recommended for further study. Let \begin{equation*}S\end{equation*} be a species tree in which \begin{equation*}xy\end{equation*} is the parent species of \begin{equation*}x\end{equation*} and \begin{equation*}y\end{equation*}. Each species, e.,g. \begin{equation*}x\end{equation*}, is assigned a set $D^x$ of domains that belong to a domain family \begin{equation*}A, B,\ldots, Z\end{equation*}. Let \begin{equation*}R^x = \{P^x_1, \ldots, P^x_k\}\end{equation*} be a partition of $D^x$ called a *domain composition family* of *domain* compositions \begin{equation*}P^x_1, \ldots, P^x_k\end{equation*}. As these are sets, the ordering of domains within a gene is ignored. Phylogenetic trees are inferred for each domain family individually. Reconciling domain trees for each domain family using the known species tree assigns domain nodes to each species node \begin{equation*}xy\end{equation*} in \begin{equation*}S\end{equation*}. The nodes which have a direct child in at least one of the descendant species are the elements of \begin{equation*}D^{xy}\end{equation*}. Let \begin{equation*}\tilde D^x\end{equation*} be the set \begin{equation*}D^x\end{equation*} relabeled in a way that each domain in \begin{equation*}x\end{equation*} receives the name of its ancestral domain in \begin{equation*}xy\end{equation*}, and let \begin{equation*}\tilde R^x\end{equation*} be the according domain composition family. If there is a duplication event in \begin{equation*}x\end{equation*}, the two domains map to the same ancestral domain in \begin{equation*}xy\end{equation*}, thus \begin{equation*}|\tilde D^x |\leq | D^x|\end{equation*}. Since \begin{equation*}R\end{equation*} is known only for the leafs, the problem is to find partitions for the inner nodes, i.e.domain composition families of ancestral species that minimize some cost

\begin{equation*}\delta(R^{xy}, \tilde R^x) + \delta(R^{xy}, \tilde R^y)\end{equation*}

which can be arbitrarily defined. Behzadi and Vingron suggest an additive measure given by 

\begin{equation*}\delta(R^{xy}, \tilde R^x) = \sum_i \delta(R^{xy}, \{\tilde P^x_i\})\end{equation*} thus we solve

 

\begin{equation*}\[\arg\min_{R^{xy}}\left\{\sum_i \delta(R^{xy}, \{\tilde P^x_i\}) + \sum_i \delta(R^{xy}, \{\tilde P^y_i\})\right\}\]\end{equation*}

 where \begin{equation*}\delta(R^{xy}, \{\tilde P^x_i\})\end{equation*} measures the number of deletions and merges that are necessary to transform the elements of \begin{equation*}R^{xy}\end{equation*}, the domain compositions of the parent, to the child domain composition \begin{equation*}\tilde P^x_i\end{equation*}. Ignore all \begin{equation*}P^{xy}_j \end{equation*} that do not contain any domain in \begin{equation*}\tilde P^x_i\end{equation*}, and store the indices of the remaining in

\begin{equation*}J:= \{j| P^{xy}_j \cap \tilde P^x_i \neq \emptyset\}\end{equation*}

The number of merges to have all domains of \begin{equation*}\tilde P^x_i\end{equation*} in one set is therefore \begin{equation*}|J|-1\end{equation*}. The resulting set could contain other domains that need to be deleted as they are not in the child domain composition; their number is

\begin{equation*}|\bigcup_{j\in J}P^{xy}_j-\tilde P^x_i| = \sum_{j\in J}| P^{xy}_j-\tilde P^x_i|\end{equation*}

Assigning costs for union and deletion yields the partitioning score

\begin{equation*}S := \delta(R^{xy}, \{\tilde P^x_i\}) = c_U \cdot \underbrace{(|J |-1)}_{\text{\# unions}} + c_D \cdot\underbrace{\sum_{j\in J}| P^{xy}_j-\tilde P^x_i|t}_{\text{\# deleted elements}}\end{equation*}

The entire tree is reconstructed in a bottom-up pass including the root.

### Heuristics for partitioning

As the number of possible partitions for domains into genes grows rapidly, complete enumeration is only possible for the simplest cases and not suitable for real applications. For BV, it was suggested to use simulated annealing to solve the partitioning problem [4]. We explored a deterministic algorithm we call weak edge erosion (see below) to find a suitable partition. 

#### Weak Edge Erosion

Weak edge erosion is a hierarchical graph clustering method based on the idea of attacking a network of affinities between elements at its weakest points and recursively create clusters by separating network components. The affinity graph is defined as follows: Consider an undirected loop-free graph \begin{equation*}G\end{equation*} with a vertex set \begin{equation*}V(G) := D^{xy}\end{equation*}. An edge between vertices \begin{equation*}v_1, v_2\end{equation*} is assigned a cost according to the number of sets in the child partitions that contain \begin{equation*}\{v_1, v_2\}\end{equation*} as a subset after relabeling. Note that each set induces a clique in this affinity graph. The edge weight measures the affinity of two vertices to occur together in a set. Thus removing edges corresponds to breaking the affinity between elements. To find a good approximation for the partitioning problem, we let $G := G_N$, store \begin{equation*}G_N\end{equation*} in a  tree node \begin{equation*}N\end{equation*},  cut the graph and store the resulting components \begin{equation*}G_i\end{equation*} in child nodes \begin{equation*}i\end{equation*} of \begin{equation*}N\end{equation*}. These nodes are processed recursively until the vertex set in a node scores 0 with the score \begin{equation*}S\end{equation*}. Then the nodes are checked in a bottom-up pass as to whether their subset or the subsets of their children score better, and this solution is passed to the parent. Cluster boundaries are induced by regions sparse in edges. But dividing a set to create partitions translates to cutting through a large number of edges in a clique, so minimal cuts and related concepts of connectivity are of limited use. Particularly, min-cut tends to separate single vertices from cliques, thus creating a suboptimal partition. To obtain meaningful cuts for our purpose, we introduce the concept of *weak edges*. Let each vertex be labeled by the sum of weights of all its incident edges. From the perspective of an edge, high vertex weights mean that the vertices have a strong connection to a set of other vertices, so edges are regarded to become weaker as their vertex weights grow. We thus define a total order of weakness: let \begin{equation*}e_m, e_n\in E(G)\end{equation*} be two edges, and \begin{equation*}v^-, v^+ \in V(G)\end{equation*} be two vertices such that \begin{equation*}w(v^-) \leq w(v^+)\end{equation*}. \begin{equation*}e_m\end{equation*} is weaker than \begin{equation*}e_n\end{equation*}, denoted by \begin{equation*}e_m \prec e_n\end{equation*}, if the corresponding edge has a lower weight:

\begin{equation*}w(e_m) < w(e_n)  \Rightarrow  e_m \prec e_n\end{equation*}

This first condition accounts for that we prefer weaker affinities to be violated. If the edge weights are equal, we want to exploit the weakening effect of high vertex weights, thus the weakness order is determined by the heavier vertices:

\begin{equation*}w(v^+_m) > w(v^+_n) \Rightarrow e_m \prec e_n\end{equation*}

If these are equal as well, the relation between the lighter vertices decides:

\begin{equation*}w(v^-_m) > w(v^-_n) \Rightarrow e_m \prec e_n\end{equation*}

The concept of edge weakness is illustrated in Figure 2. As the cost function is additive, we can find an approximate solution to the partitioning problem by splitting \begin{equation*}D^{xy}\end{equation*} into nested \begin{equation*}P^{xy}_N\end{equation*} recursively and combine the local results. We use Algorithm 1 to find a good partition \begin{equation*}R^{xy}_N\end{equation*} of \begin{equation*} V(G) = D^{xy}\end{equation*} with a cost \begin{equation*}C_N\end{equation*}. The weakest edges are removed until the graph is decomposed into two or more components. A cut tree of \begin{equation*}G\end{equation*} is built such that a node contains \begin{equation*}G\end{equation*} and its children contain the connected components. See Figure 3 for an example.

**Figure:**
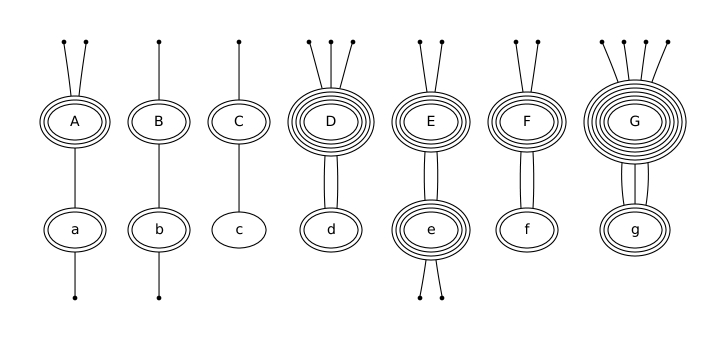
**Figure 2.** **Example of edge weakness.** Edges are sorted from left to right in decreasing order of weakness. Weights are depicted by the multiplicity of lines. The edge \begin{equation*}(A,a)\end{equation*} has a weight of 1, node \begin{equation*}A\end{equation*} has a combined weight of incident edges of 3, and a of 2. The upper row contains  the heavier nodes incident to the central edge. The lower the edge weight, the weaker this edge, e.g.  

\begin{equation*}w(C,c) < w(D,d) \Rightarrow (C,c)\prec (D,d)\end{equation*}

If edges tie, such as \begin{equation*}(A,a)\end{equation*}, \begin{equation*}(B,b)\end{equation*} and \begin{equation*}(C,c)\end{equation*}, the heavier nodes decide:  

\begin{equation*}w(A) > w(B) \Rightarrow (A,a) \prec (B,b)\end{equation*} \begin{equation*}w(A) > w(C) \Rightarrow (A,a) \prec (C,c)\end{equation*} 

If the upper nodes tie as well, as for \begin{equation*}w(B) =w(C)\end{equation*}, the weights of the lighter nodes decide:  

\begin{equation*}w(b) > w(c) \Rightarrow (B,b) \prec (C,c)\end{equation*}

 Walking through the cut-tree in a bottom-up procedure, we decide whether the block in the parent node or the two set blocks in its children yield a better score \begin{equation*}S\end{equation*}, and pass this local solution upwards until the root contains an approximate solution for the partitioning problem.

**Figure:**
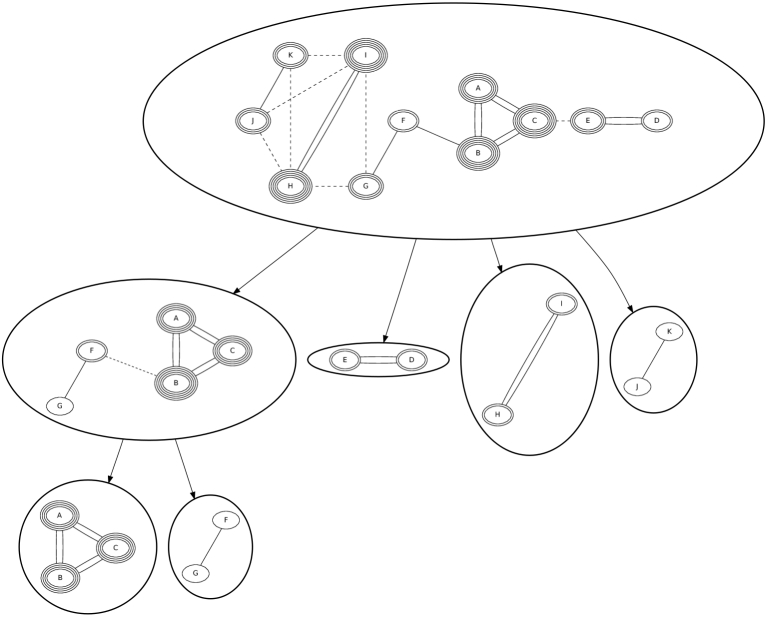
**Figure 3. A worked example of a cut tree for the sets**  

\begin{equation*}\{A,B,C\}\end{equation*}, \begin{equation*}\{A,B,C\}\end{equation*}, \begin{equation*}\{C,E\}\end{equation*}, \begin{equation*}\{D,E\}\end{equation*}, \begin{equation*}\{D,E\}\end{equation*}, \begin{equation*}\{B,F\}\end{equation*}, \begin{equation*}\{F,G,H,I\}\end{equation*}, \begin{equation*}\{H,I,J,K\}\end{equation*} 

Each node represents a set block of the vertices it contains. Vertex weights are denoted by the number of peripheries. Note that this weight changes during the process according to the sum of incident edges (e.g. in vertex \begin{equation*}I\end{equation*}). The weakest edges that are attacked during an erosion are denoted by dashed lines. Final erosions of the remaining cliques in the leafs are omitted, since they do not yield a better score. In the root, the weakest edges are \begin{equation*}\{F,H\}\end{equation*} and \begin{equation*}\{F, I\}\end{equation*}, as their weights are 1, \begin{equation*}I\end{equation*} and \begin{equation*}H\end{equation*} have the maximum number of incident edge weights:  

$w(v^+) = w(I) =w(H) = 6$

and \begin{equation*}F\end{equation*} is the maximum lighter vertex adjacent to \begin{equation*}I\end{equation*} and \begin{equation*}H\end{equation*}: 

\begin{equation*}w(v^-) = w(F) = 4\end{equation*}

However, this does not suffice to disconnect the graph, so vertex weights are recalculated: 

\begin{equation*}w(H) = w(I) = 5\end{equation*}, \begin{equation*}w(F) = 2\end{equation*} 

and all edges with a weight of 1 are removed, \begin{equation*}w(v^+) = 5\end{equation*} and \begin{equation*}w(v^-)=3\end{equation*}. Relying on min-cut instead of erosion would fail to create set blocks \begin{equation*}\{F, G\}\end{equation*}, \begin{equation*}\{H, I\}\end{equation*} and \begin{equation*}\{J, K\}\end{equation*}, as it would cut off \begin{equation*}G\end{equation*}, \begin{equation*}J\end{equation*} and \begin{equation*}K\end{equation*} as single vertices.

 The complete procedure is summarized in Algorithm 1:

**Figure:**
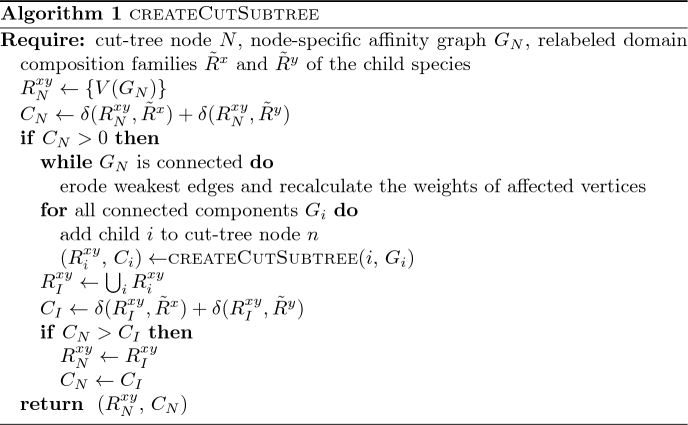

#### Simulated annealing

The original authors of BV propose simulated annealing to solve the underlying partitioning problem [8]. We implemented a dynamic cooling schedule, which sets most parameters automatically [9]. Starting from a valid domain configuration, the fusion and fission of groups of domains and the swap of individual domains are used to generate related domain compositions. The simulated annealing procedure is then used to minimize the score \begin{equation*}S\end{equation*} of the domain composition.

### Identification and curation of implausible domain trees

Due to their short length, the inferred domain phylogenies often disagree with each other and the species tree. The BV algorithm was proposed for ideal data and does not consider errors in the underlying domain topologies. A practical consequence of such errors are that the order of speciation and duplication events between adjacent domains do not agree. These conflicts can lead to duplicate nodes in the reconstructed composition, for example nodes that have successors within the same protein. The BV algorithm will then produce MDPs with a high partition score due to additional copies of the conflicting domains. Our algorithm aims to produce a partition with an improved score by applying nearest neighbor interchanges on the conflicting domain trees. For each modification the ancestral composition is reconstructed and the modification with the lowest score \begin{equation*}S\end{equation*} is used as a correction.  

### Simulation of MDP phylogenies

Species trees were generated following the Yule-Harding model. An initial domain composition of three families in a single protein was placed in the root and passed to its children, whose protein domain compositions underwent evolutionary events of genes (fusion, duplication) and domains (gain, duplication, loss). Subtree pruning and regrafting (SPR) operations were applied on the domain trees to create perturbed input data. 

### Construction of domain phylogenies

For an empirical evaluation we ran global models of the PFAM database [10] with HMMER's hmmpfam and hmmalign [11] against the UniProt/Swiss-Prot database [12] to identify domains and construct alignments. Maximum Likelihood trees were inferred from the alignments using PhyML [13]. The trees were rooted using Notung [14].

## Results and Discussion

As we cannot obtain true ancestral multi-domain protein compositions, we relied on simulations to test the validity of the algorithm and to inspect its performance when errors are introduced. The practical performance was evaluated on known instances of MDPs, available on the accompanying website. A brief, non-trivial example is presented in Fig. 1.

### Simulations

To assess the performance of the algorithm on controlled input, species and domain phylogenies were simulated. The reconstructed domain compositions were compared to the original compositions using the partition distance measure, which ranges between zero and the size of the compositions [15].  Most ancestral partitions can be reconstructed perfectly but not all events can be mapped correctly. For example, the gain of a new domain family and the subsequent loss in one of its immediate children cannot be reconstructed accurately. The high standard deviation suggests that there are a few compositions which differ very much from their simulated counterpart. SPR operations lower the quality of the reconstruction.

**Table:** 

Taxa	0 SPR	1 SPR	2 SPR	5 SPR	10 SPR
10	0.06 (0.35)	0.10 (0.40)	0.13 (0.45)	0.23 (0.66)	0.34 (0.86)
30	0.09 (0.47)	0.11 (0.50)	0.13 (0.52)	0.16 (0.55)	0.22 (0.65)
50	0.09 (0.46)	0.09 (0.46)	0.10 (0.48)	0.13 (0.50)	0.16 (0.54)

**Table 1.** Average partition distance and standard deviation of reconstructed domain compositions. The simulated trees had 10, 30 and 50 taxa and up to ten SPR operations were applied. The distance was calculated between simulated and reconstructed domain compositions of all ancestral species and normalized by the number of taxa. Each experiment was repeated 50 times.

An automated correction of incongruent domain phylogenies is effective for cases with minor errors. Manual curation is advised if domain phylogenies cannot be inferred reliably. To this end, internal nodes with high scores are tagged for assessment.

### Performance

The run times of our implementation are short. We simulated 50 replicates of input data containing 50 taxa and no regrafting operations and ran the implementation under Linux on an AMD 64 X2 3200+ processor. The erosion heuristic inferred a MDP reconstruction in 2.06s±0.25s, while the simulated annealing procedure took 153s±22s to achieve comparable solutions. In practice, the calculation of reliable domain families bounds the performance. 

### Application

JMJ domains are found in proteins involved in chromatin remodeling complexes often together with domain families such as ARID, PHD and PLUN-1 [16]. They provide a concise example, parts of which are presented in Fig. 1. Only three species were selected for display; using a larger number of species is advisable for the inference of individual domain phylogenies. 

## Conclusion

Our solution for the inference of phylogenies of multi-domain proteins provides a simple and easy-to-use interface. The weak edge erosion heuristic provides considerable speed-up over simulated annealing while maintaining comparable solution quality. Beyond the application on MDPs, such methods could be applied to reconstruction of partial homologous units such as bacterial operons or protein complexes. Future work will be directed at improving the quality of the tree reconciliation. 

## Availability and requirements

The program was successfully tested under Python 2.6 and 2.7 on Windows, Mac OS X and Linux. It receives input for species and domain trees as well as parameters in Nexus format. The output can be visualized using GraphViz. The source code with additional figures and examples can be found at http://virulence.molgen.mpg.de/cocos/ and is freely available under a BSD license.    

## Author contributions

Implementation: MH and JW. Weak edge erosion: JW. Domain analysis: ST, CS, RK. Example data: IK, CS. Simulations: MH and RK. Wrote the paper: MH, JW and RK.  

## Financial support

The authors have no support or funding to report.

## Competing interests

The authors have declared that no competing interests exist.
